# Expression of Shelterin Component *POT1* Is Associated with Decreased Telomere Length and Immunity Condition in Humans with Severe Aplastic Anemia

**DOI:** 10.1155/2014/439530

**Published:** 2014-05-06

**Authors:** Ting Wang, Shu-chong Mei, Rong Fu, Hua-quan Wang, Zong-hong Shao

**Affiliations:** Department of Hematology, General Hospital, Tianjin Medical University, 154 Anshandao, Heping, Tianjin 300052, China

## Abstract

Abnormal telomere attrition has been found to be closely related to patients with SAA in recent years. To identify the incidence of telomere attrition in SAA patients and investigate the relationship of telomere length with clinical parameters, SAA patients (*n* = 27) and healthy controls (*n* = 15) were enrolled in this study. Telomere length of PWBCs was significantly shorter in SAA patients than in controls. Analysis of gene expression of Shelterin complex revealed markedly low levels of *POT1* expression in SAA groups relative to controls. No differences in the gene expression of the other Shelterin components—*TRF1*, *TRF2*, *TIN2*, *TPP1*, and *RAP1*—were identified. Addition of IFN-***γ*** to culture media induced a similar fall in POT1 expression in bone marrow cells to that observed in cells cultured in the presence of SAA serum, suggesting IFN-***γ*** is the agent responsible for this effect of SAA serum. Furthermore, ATR, phosphorylated ATR, and phosphorylated ATM/ATR substrate were all found similarly increased in bone marrow cells exposed to SAA serum, TNF-***α***, or IFN-***γ***. In summary, SAA patients have short telomeres and decreased POT1 expression. TNF-***α*** and IFN-***γ*** are found at high concentrations in SAA patients and may be the effectors that trigger apoptosis through POT1 and ATR.

## 1. Introduction

Telomeres are located at the end of linear eukaryotic chromosomes and consist of repetitive TTAGGG DNA sequences and specific interacting proteins that together form a capping structure that prevents chromosomal damage and degradation [1]. A small amount of telomere length is normally lost during each cell division, with telomere homeostasis being a complex process affected by both intrinsic and extrinsic factors. These include heredity, epigenetics, and environmental factors such as inflammation and stress [2]. Telomere length can be influenced directly by the telomerase enzyme and indirectly by a group of proteins known as the Shelterin complex.

Aplastic anemia (AA) is a potentially life-threatening failure of hemopoiesis characterized by pancytopenia and hypocellular bone marrow. Other than those patients with inherited conditions, most cases are acquired and immune-mediated [3]. Shortened telomeres were first identified in AA patients more than a decade ago [4], with about one-third to one-half of AA patients possessing short telomeres. Those with the shortest telomeres appear to have had longer disease duration and were more likely to develop latent malignant clonal changes. Several studies have identified telomere length improvement with disease recovery, while patients with irreversible telomere attrition typically have poor outcomes [5, 6]. Our department treats many patients with severe aplastic anemia (SAA). In this study, our first aim was to identify the incidence of telomere shortening in SAA patients and to ascertain whether telomere length correlates with clinical parameters.

Previous studies have determined that most family members of AA patients with short telomeres have deficiencies in bone marrow hematopoietic function. AA with telomere attrition has therefore been recognized as a genomic disease and stem cell transplant is currently the only effective treatment available. Many AA patients with short telomeres carry mutations in genes such as those encoding the telomerase components* TERT* or* TERC*, the genes* NOP10* and* NOLA1*, and the genes of Shelterin components [7–9]. However, the rate of mutations within these genes is insufficient to explain the high incidence of shortened telomeres in AA. It has been reported that the proportion of AA cases with* TERC* and* TERT *mutations is about 5–10 percent, with mutations in other genes such as those of Shelterin components rare and predominantly found in pediatric cases [10]. Therefore, in addition to genetic mutations, we speculate that other mechanisms lead to telomere attrition in AA. In this study we focus on the Shelterin complex and POT1 in particular.

The Shelterin complex interacts directly with telomeric DNA and mediates telomere homeostasis by allowing access to both positive and negative regulators of telomere length that use both telomerase-dependent and telomerase-independent mechanisms [11]. In this study, we further investigate expression of Shelterin complex components at different stages of SAA progression from newly detected disease to posttreatment and probe for clues as to how telomere attrition and Shelterin abnormalities could trigger SAA.

## 2. Materials and Methods

### 2.1. Patients and Controls

Peripheral blood and bone marrow samples from SAA patients and age matched controls were obtained after written informed consent had been received in accordance with the Declaration of Helsinki. SAA diagnosis was established by bone marrow biopsy, bone marrow smear, and peripheral blood cell count. SAA was defined by the following criteria: (1) bone marrow cellularity <25% or 25–50% with <30% residual (hematopoietic) cells; (2) two of the following three: neutrophils <0.5 × 10^9^/L, platelets <20 × 10^9^/L, and reticulocytes <20 × 10^9^/L; (3) no malignant cell infiltration, no extensive marrow fibrosis, no extensive iron deposition, no evidence of malignant disease, and no myelofibrosis, storage disease, or chemotherapy.

Recovering SAA patients were those who improved after therapy with antithymocyte globulin, cyclosporine, glucocorticoid, and hematopoietic stimulating factors (recombinant human erythropoietin, granulocyte colony-stimulating factor, recombinant human thrombopoietin, and/or IL-11 in combination). All recovering patients achieved bone marrow hematopoietic recovery, which was defined as substantial improvement in 2-3 lineages. Some patients became transfusion independent while some had normal peripheral blood cell counts but still required drug therapy.

### 2.2. Measurement of Telomere Length

DNA was extracted from 1-2 × 10^6^ target cells by a DNA extraction kit (Tian'enze Biotech, Beijing, China). Mean length of terminal restriction fragments (TRF) was measured using the TeloTAGGG telomere length assay kit (Roche). Purified DNA (1-2 *μ*g) was digested with* HinfI/RsaI *mixture. Following electrophoresis and transfer, the membrane containing DNA was hybridized to digoxigenin-labeled probe specific for telomeric DNA repeats. After incubation with a digoxigenin-specific antibody, TRF length was visualized by a highly sensitive chemiluminescent reagent in the kit by gel imaging analysis system (Genesnap G, Gene Company, American). Overall mean TRF length was determined using Gene Tools software.

### 2.3. RNA Isolation and qPCR

PBMCs were lysed (1-2 × 10^6^ target cells) in TRIzol reagent (Invitrogen, USA). Total RNA was isolated according to the manufacturer's protocol (Invitrogen). Total RNA was dissolved in RNase-free water and quantified using a UV spectrophotometer (NanoDrop ND-1000, Thermo Scientific, USA). Equal amounts of RNA were reverse transcribed using the High-Capacity Reverse Transcription kit (TianGen Biotech, China) according to manufacturer's protocol. Real-time PCR was performed with 2 *μ*L of each cDNA working solution in a final volume of 20 *μ*L containing 10 *μ*L 2x RealMasterMix/20x SYBR solution (TianGen Biotech) and 300 nM of each sense and antisense primer. PCR was carried out in a Bio-Rad PCR iQ5 (Bio-Rad, USA) using the following thermal cycling profile for all genes of interest: 95°C for 15 min, followed by 40 cycles of amplification (95°C for 10 s, indicated annealing temperature for 30 s, 72°C for 32 s, and 55°C for 10 s). Absorption values of the SYBR Green I in each tube were detected at the end of each cycle. A melting curve analysis of PCR products from 55 to 95°C was also performed after PCR amplification. Primer sequences and annealing temperature are listed in Table 1. The relative expression level of genes of interest was calculated by the ΔΔ*C*_*t*_ method (User Bulletin number 2, ABI PRISM 7700 Sequence Detection System).

### 2.4. Cell Culture

3 × 10^6^ of target bone marrow mononuclear cells (BMMNCs) were maintained in four different cell culture conditions: Group 1: RPMI1640 (Invitrogen, USA) supplemented with 10% fetal bovine serum (FBS, HYCLONE, USA); Group 2: 1640 medium supplemented with 10% SAA serum; Group 3: 1640 medium supplemented with 10% FBS and 100U/mL TNF-*α*; Group 4: 1640 medium supplemented with 10% FBS and 100U/mL IFN-*γ*. The cultures were incubated at 37°C in humidified 95% air with 5% CO_2_ for 24 hours_._

### 2.5. Flow Cytometry for Apoptosis Analysis

Apoptosis was detected using a commercially available Annexin V apoptosis detection kit (Becton Dickinson, USA) and flow cytometry. Following 24 h incubation under indicated culture conditions, cultured cells from groups 1 and 2 were harvested, washed with ice-cold phosphate-buffered saline (PBS), and incubated with HEPES buffer. Cells were washed with binding buffer and then analyzed with a FACSCalibur flow cytometer (Becton Dickinson). Approximately 20,000 counts were made for each sample.

### 2.6. Immunoblotting

Cultured cells were collected and lysed directly in lysis buffer supplemented with complete protease inhibitor cocktail (Roche, Switzerland) and phosphatase inhibitors (Roche, Switzerland). Protein levels were quantified using the BCA method. Proteins were subjected to SDS-PAGE and transferred onto a nitrocellulose membrane. Membranes were blocked with confining liquid (TianGen Biotech) and then probed with primary antibody solution. Primary antibodies, ATR, Phospho-ATR Ser428, and Phospho-Ser/Thr ATM/ATR substrate (Cell Signaling Technology, USA), were diluted with PBS to a final concentration of 1 : 10,000.

Incubation with relevant secondary antibodies (Zhongshan Biotech, China) was performed in PBS and visualization was achieved using Super ECL Plus Detection Reagent.

### 2.7. Statistical Analysis

All statistical calculations were performed using SPSS Statistics 15.0. Between-group differences were investigated by analysis of covariance (ANCOVA). Spearman's rank correlation test was used to calculate the correlation between telomere length and clinical data. Nonparametric tests were used when data was nonnormal distribution. Pre- and postlab data were compared by paired *t*-test. Statistical significance was accepted if *P* < 0.05.

## 3. Results

### 3.1. Clinical and Demographic Parameter of Patients Participating in the Study

Twenty-seven SAA patients (age range 5–69 years) and 15 normal controls (age range 5–67 years) were enrolled in the present study. Within the SAA cohort, there were nine untreated and 18 recovering patients. Sequential samples from the same patient were excluded from the analysis.  Table 2 details patient information.

### 3.2. Telomere Attrition of PWBCs in SAA and the Relationship between Telomere Length and Clinical Parameters

**Figure 1:**
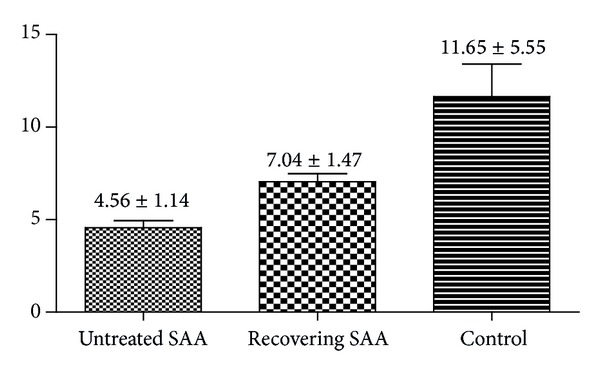
Telomere length of PWBCs.

Telomere length in peripheral white blood cells (PWBCs) was found to be significantly shorter in untreated SAA patients (4.89 ± 1.66 kb) and recovering SAA patients (7.04 ± 1.47 kb) than in control patients (11.65 ± 5.55 kb, Figure 1). No significant difference in telomere length was detected between untreated SAA and recovering SAA patients. As expected, telomere length in PWBCs of healthy controls declined progressively with age (*r* = −0.748; *P* = 0.013). However, telomere length in SAA patient PWBCs was age independent, with no statistically significant age-related trend found in either the SAA untreated group (*r* = −0.086; *P* = 0.77) or the recovering group (*r* = −0.433; *P* = 0.244).  Table 4 details ages and telomere length of both patients and controls.

**Figure 2:**
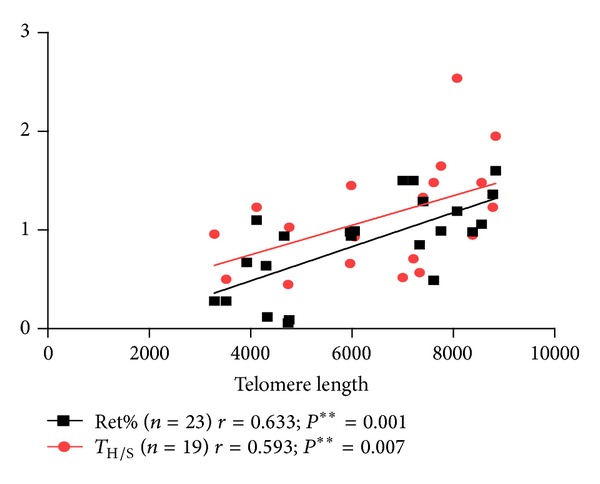
Relationship between TL and T_H/S_ and TL and Ret%.

**Figure 3:**
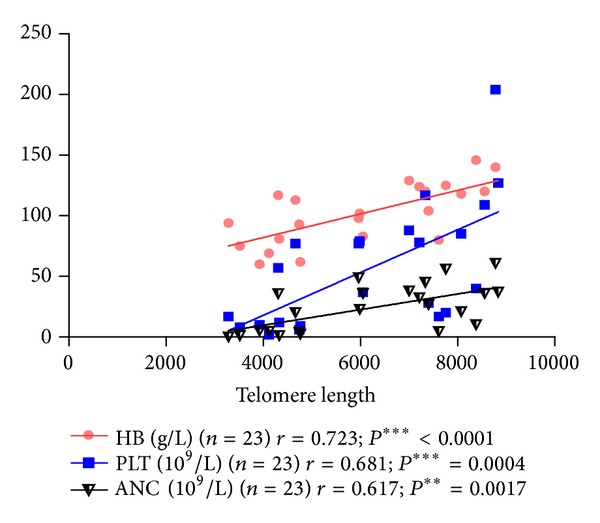
Relationship between hemoglobin, platelet count, absolute neutrophil count, and telomere length. HB: hemoglobin. PLT: platelet count. ANC: absolute neutrophil count.

Unexpectedly, shortened telomere length correlated with low hemoglobin (*r* = 0.732; *P* < 0.0001), platelet count (*r* = 0.681; *P* = 0.0004), absolute neutrophil count (*r* = 0.617; *P* = 0.0017), and proportion of reticulocytes in peripheral blood (*r* = 0.633; *P* = 0.001). Furthermore, the ratio of CD4+ T-helper lymphocytes to CD8+ T-suppressor (T_H/S_) was found to correlate with the telomere length of PWBCs (both telomere length and T_H/S_ were measured in 19/27 patients; *r* = 0.593; *P* = 0.007; Figures 2 and 3). However, if untreated and recovering patients were divided into separate groups no relationship between the telomere length and the above clinical parameters was apparent.

There was no relationship between the telomere length of PWBCs in SAA patients and the absolute value of the lymphocyte count (*r* = 0.137; *P* = 0.599); the recovery time of at least one lineage of bone marrow hematopoietic cells (*r* = 0.261; *P* = 0.280); the recovery time of three lineages of bone marrow hematopoietic cells (*r* = 0.253; *P* = 0.363); the duration of disease (*r* = 0.193; *P* = 0.378); the clone of paroxysmal nocturnal hemoglobinuria (PNH) cells (*r* = −0.14, *P* = 0.525); or the chromosomal abnormalities (*r* = −0.257; *P* = 0.236).

### 3.3. mRNA Expression Abnormalities of Shelterin Complex Components in PWBCs from SAA Patients

**Figure 4:**
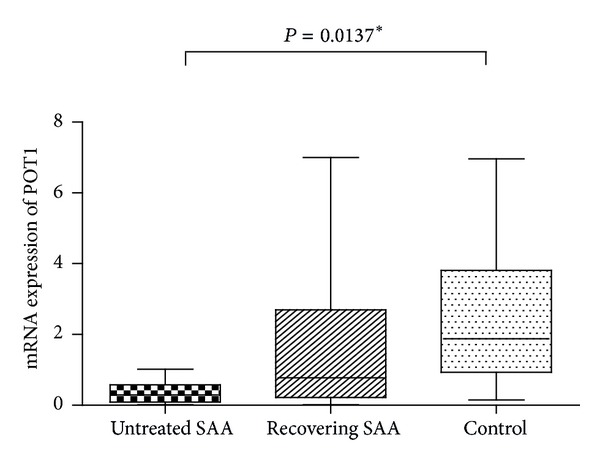
POT1 mRNA expression in different groups.

Mammalian telomeres are associated with Shelterin, a protein complex that functions to protect DNA ends from being recognized as double strand breaks that would trigger a DNA damage response [12, 13]. Proteins comprising the Shelterin complex are TRF1, TRF2, TIN2, RAP1, TPP1, and POT1. While each protein has been reported to participate in telomere synthesis and telomere protection, little is known about any potential role for them in AA. We measured the levels of mRNA of these genes in our patient samples and identified significantly lower expression of* POT1* in the SAA untreated group (0.29, range: 0.09–0.58) relative to the recovering group (0.78, range: 0.22–2.69) and the control group (1.88, range: 0.93–3.81; *P* = 0.0137; Figure 4). No significant differences in the expression level of* TRF1, TRF2, TIN2, TPP1, *or* RAP1* between groups were detected (Table 3).

Though low expression of POT1 was significantly associated with short telomeres (*r* = 0.398; *P* = 0.022*), there was no significant relationship between POT1 expression and T_H/S_ (*r* = 0.168; *P* = 0.467).

### 3.4. High Apoptosis Rate and Expression of ATM/ATR Proteins in SAA Serum Cultured Normal BMMNCs

**Figure 5:**
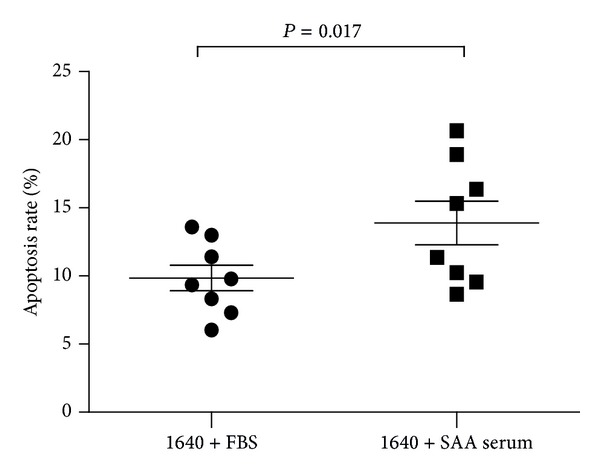
Normal BMMNCs cultured with SAA serum undergo a higher apoptosis rate than those cultured with FBS.

High levels of apoptosis of hematopoietic cells in SAA patients have been previously reported, with high levels of TNF-*α* and IFN-*γ* suspected to contribute to this phenomenon [14]. Our research team has also detected high levels of these cytokines in SAA patient serum in the past. TNF-*α* and IFN-*γ* reduce the numbers of human hematopoietic progenitor-derived colonies in vitro and efficiently induce apoptosis in CD34 target cells, at least partially through the Fas-dependent cell death pathway [15]. We cultured BMMNCs from eight normal control group patients and compared rates of apoptosis when these cells were cultured in media containing SAA serum or FBS. As expected, cells cultured with SAA serum underwent significantly higher levels of apoptosis (13.88 ± 4.55) than those with FBS (9.85 ± 2.67; *P* = 0.017; Figure 5).

**Figure 6:**
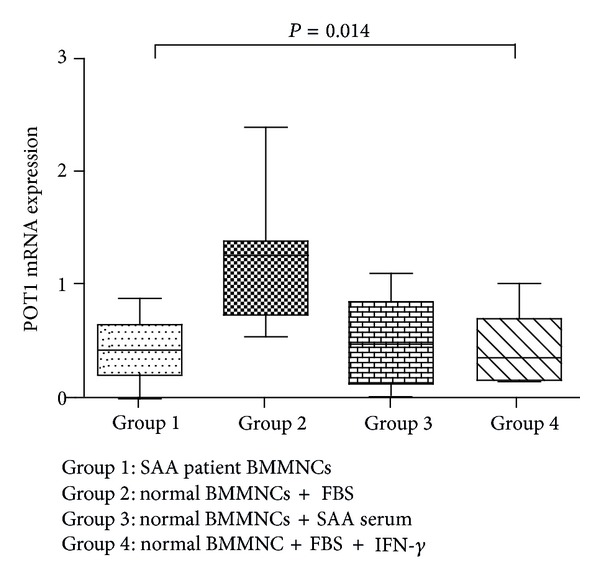
POT1 mRNA expression in normal BMMNCs is reduced in the presence of SAA serum or IFN-*γ*.

We next examined whether TNF-*α* or IFN-*γ* was responsible for the decreased mRNA expression of POT1 identified in SAA patients. We chose eight normal control and eight untreated patients at random, isolated their BMMNCs, and cultured these normal BMMNCs in media containing SAA serum. POT1 mRNA expression in normal BMMNCs cultured with FBS was markedly higher (1.17 ± 0.57) than that in cells from SAA patients (0.45 ± 0.27; these cells were harvested directly and not subjected to culture conditions), normal BMMNCs cultured with SAA serum (0.49 ± 0.38), and those cultured with 100U/mL IFN-*γ* (0.40 ± 0.35; Figure 6). Data for cells cultured in the presence of 100U/mL TNF-*α* has been omitted as the majority (5/8) had zero POT1 expression. Taken together, these data suggest that a constituent of SAA serum—maybe TNF-*α*, IFN-*γ*, or others—effectively reduced* POT1* mRNA expression in vitro as it does in SAA patients in vivo.

**Figure 7:**
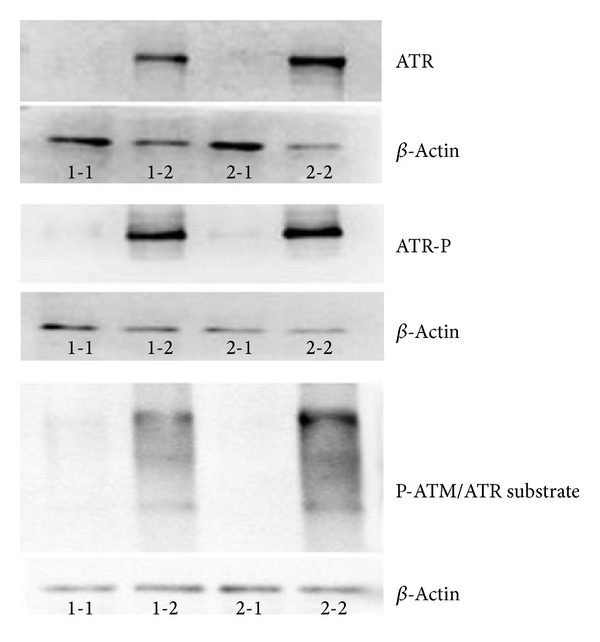
Culture of BMMNCs with media containing SAA serum increases activation of ATR. Legend: Samples 1-1 and 2-1: BMMNCs from two individual control patients cultured with 1640 media + 10% FBS. Samples 1-2 and 2-2: BMMNCs from the same two individual control patients cultured with 1640 media + 10% SAA serum.

DNA damage initiates signal transduction pathways, known as checkpoints, which culminate in cell cycle arrest. Damaged mammalian telomeres can activate the kinases ATM and ATR. The Shelterin complex represses these two important DNA damage sentinels, preventing activation of DNA damage pathways. TRF2 is able to bind to and suppress ATM, whereas POT1 prevents activation of ATR [16]. Here, we have shown that the culture of BMMNCs in media containing SAA serum or IFN-*γ* reduced expression of* POT1*. Therefore, we next determined the activation status of ATR in BMMNCs isolated from two normal individuals and cultured in the presence of FBS or SAA serum by measuring ATR protein levels, phosphorylation status, and activation of ATM/ATR substrates. BMMNCs cultured in FBS have very low levels of ATR, phosphorylated ATR, and phosphorylated ATM/ATR substrate; in contrast those cultured with SAA serum display a high level of these proteins (Figure 7).

**Figure 8:**
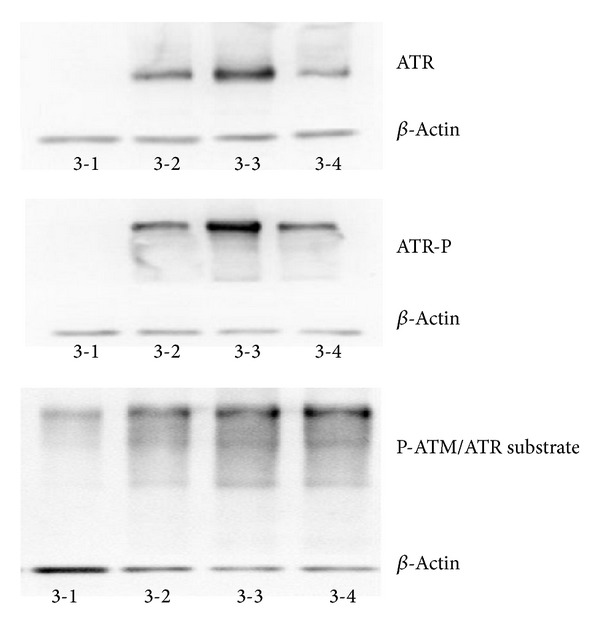
TNF-*α* and IFN-*γ* increase activation of ATR in BMMNCs. Legend: Sample 3-1: BMMNCs + 1640 media + 10% FBS. Sample 3-2: BMMNCs + 1640 media + 10% FBS + 100U TNF-*α*, Sample 3-3: BMMNCs + 1640 media + 10% FBS + 200U TNF-*α*, and Sample 3-4: BMMNCs + 1640 media + 10% FBS + 100U IFN-*γ*.

We next determined the levels of ATR, phosphorylated ATR, and phosphorylated ATM/ATR substrate in normal BMMNCs cultured with FBS and TNF-*α* or IFN-*γ*. ATR, phosphorylated ATR, and phosphorylated ATM/ATR substrate were all found to be significantly increased in cells cultured with TNF-*α* and IFN-*γ* when compared with those cultured with FBS alone (Figure 8).

## 4. Discussion

Acquired AA is prototypical of human bone marrow failure. The incidence in Western countries is two cases per million per year while in Asia this number is approximately two- to threefold higher. In China, the incidence is increased even higher with 7.4 cases per million per year [17–19]. Affected patients always have severe anemia, bleeding and infection, and a poor survival rate. Though immunosuppressive therapy and hematopoietic stem cell transplantation (HSCT) have well improved the treatment effect of AA, 30% of patients do not respond favorably to treatment and may die soon after diagnosis because of heart failure, profuse hemorrhage, or overwhelming infection [20].

The precise pathophysiology of AA is unclear, with researchers predicting antigen discovery and genetic susceptibility as the two major directions for gaining a greater understanding of the disease. Other than in its response to bone marrow-suppressive agents (such as benzene and chloramphenicol), many studies have reported SAA to behave like an immune-mediated disease. Some as-yet unknown antigens can activate T cells to secrete marrow-suppressive cytokines (e.g., TNF-*α* and IFN-*γ*), which attack bone marrow and induce apoptosis in hematopoietic progenitor cells. These cytokines induce apoptosis through the Fas-dependent and perforin pathways of cell death [15, 21–23].

Much research has focused on the cytogenetic abnormalities of AA. Some bone marrow failure patients previously diagnosed with AA received immunosuppressive therapy but had a poor response and were later found to carry repeatable gene mutations. These patients were subsequently diagnosed with Fanconi anemia, dyskeratosis congenita, Diamond-Blackfan anemia, or Shwachman-Diamond syndrome. HSCT may be chosen as a more suitable initial treatment for these diseases [3, 24, 25]. Short telomeres were identified in both the hereditary diseases above and the acquired AA. Patients with shorter telomeres (and mostly without any known telomerase mutation) had severe pancytopenia, a poor clinical response to treatment, higher possibility of relapse, an increased risk to evolve to myelodysplasia or acute myeloid leukemia, and poorer overall survival in comparison to patients with longer telomeres [26, 27]. In this study, we chose only SAA patients as subjects and found that untreated SAA patients had the shortest telomere length when compared with recovering patients and controls (77.8% of untreated patients had a mean telomere length shorter than 5 kb compared with only 14.3% of recovering patients). These findings demonstrate that telomere length is regained as disease recovery progresses. In patients with AA, helper and effector T cells are unbalanced; the ratio of helper/effector T cells is always very low, which is considered as a marker of the immune status of AA patients [28, 29]. In the present study, telomere length of PWBCs was found to correlate with T_H/S_, which means telomere length is closely related to patients' immune status. AA patients' conditions are more critical with lower hemoglobin, platelets, neutrophils, and reticulocytes. Our results indicated that the telomere length correlates with the level of HB, PLT, ANC, and Ret%, which means the telomere length is closely related with the severity of patients' condition.

Short telomeres are found in one-third to one-half of AA patients, but mutations in genes encoding the telomerase components occur in less than 10% of cases [10]. Furthermore, mutations in Shelterin components are even rarer. Therefore, we did not detect the mutation status of genes encoding telomere-related proteins; rather we measured their expression. Telomeres are regulated by Shelterin, which plays a key role in telomere capping and telomerase regulation. POT1 has a critical role in all these activities [30]. POT1 blocks telomerase from acting by binding to the 3′ end of telomeric DNA. However, when telomeres become dysfunctional, their ends are sensed as double-stranded DNA breaks. This recognition results in the activation of DNA damage checkpoints that trigger senescence or apoptosis. POT1 inhibits the activation of the ATR kinase, a DNA damage responder that can induce both apoptosis and cell cycle arrest upon activation [31–36].

In the present study, we detected very low POT1 mRNA expression in patients with SAA. However, as POT1 is a competitive inhibitor of telomerase, telomerase in SAA patients should have a greater opportunity to elongate telomeres. Why then do SAA patients have short telomeres? Recently research demonstrates that proinflammatory cytokines including TNF-*α* and IFN-*γ* inhibit telomerase activity [37–39]. Moreover, in vitro research using various cell types indicates that an accumulation of senescent cells with critically short telomere may produce proinflammatory factors, which could in turn contribute to an increased inflammatory load [40–42]. SAA is a classic example of a disease that triggers T cells to release a great quantity of marrow-suppressive cytokines, ultimately leading to bone marrow failure. We suspect that these cytokines contribute to a poor environment, leading to both low POT1 expression and telomerase suppression. These two events occur simultaneously at the beginning of SAA and could explain why telomeres in SAA patients are shortened despite a reduction in POT1-mediated telomerase inhibition.

**Figure 9:**
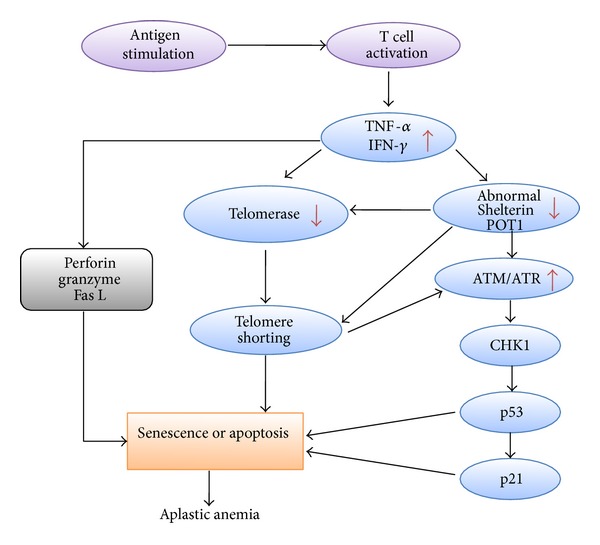
Postulated pathogenesis of aplastic anemia.

Once telomeres are critically short, they become dysfunctional and are recognized as double-stranded DNA breaks. This involves activation of ATM, which is normally suppressed by TRF2 and/or activation of ATR which is normally suppressed by POT1 [43]. In SAA patients, the low expression of POT1 results in failure of ATR inhibition. Following ATR activation, cells enter either senescence or apoptosis pathways. In the third part of our study, we showed that both SAA serum and marrow-suppressive cytokines can inhibit POT1 mRNA expression and increase the expression of ATR, phosphorylated ATR, and the substrate of ATM/ATR. We therefore describe AA pathogenesis in a new figure, incorporating our novel findings and those of other recent studies (Figure 9).

## 5. Conclusion

The telomeres of SAA patients are severely shortened, with shortening correlated with the degree of illness. TNF-*α* and IFN-*γ* are found at high concentrations in SAA and may be responsible for triggering senescence or apoptosis through* POT1* and activation of ATM/ATR. Furthermore, SAA patients who possess short telomeres could also benefit from immunosuppressive therapy.

## Figures and Tables

**Table 1:** Target gene primer sequences and annealing temperature.

Gene	Primer (5′ → 3′)	Annealing temperature (°C)
*β*-Actin	F CCTCATGCCATCCTGCGTCTG R TTGCTCGAAGTCTAGGGCAACATAG	58
TRF1	F AAGTCCTGAAAGCCCTGAATC R TTCCTGTGCCTCCAAAATCT	58
TRF2	F CTGTATTCATTTTGCTAACTTAG R ATTCTTAACACTCTCTAGAGTTG	55
TIN2	F CTTCATTCCTACTAAACTACTTG R ACTGTAGAGACAGTTCTAGACCT	57
POT1	F CAGAAAAGTGTGGATATG R AAGTAAAAGAAGTGTGGG	55
TPP1	F GCGTGACGTCTCACATCG R ATCGTGATGGTTCTGCCTTC	55
RAP1	F TGTGGTGTGTGTGTGTGTGG R CCCCACACACACCACACAC	56

**Table 2:** Clinical and demographic parameters of patients participating in the study.

Diagnosis	Number	Age (y)	Duration (mo)	ANC (/mm^3^)	Hb (g/dL)	Plts (10^3^/mm^3^)	Ret%	Therapy
Untreated SAA	9	33 (5–69)	2 (1–4)	0.31 ± 0.20	82.11 ± 20.01	11.22 ± 5.85	0.45 ± 0.39	Not previously treated except for transfusions
Recovering SAA	18	20 (5–48)	24 (6–120)	3.33 ± 1.22	118.94 ± 16.78	89.35 ± 43.08	1.14 ± 0.27	Treated with combination indicated
Recovering group before combination treatment except transfusion	18	18 (4–47)	Unable to be determined for some patients	0.21 ± 0.15	72.12 ± 23.58	9.71 ± 4.89	0.25 ± 0.20	Not previously treated except for transfusions

Values are mean ± SEM. Age and duration values are mid (min, max). ANC: absolute neutrophil count; Hb: hemoglobin; Plt: platelet; Ret: reticulocyte.

**Table 3:** Shelterin complex mRNA expression in PWBCs [*x* ± *s*, M (*P*_25_–*P*_75_)].

Gene	Untreated group (*n* = 9)	Recovering group (*n* = 17)	Control group (*n* = 15)	*P* value
POT1	0.29 (0.09–0.58)	0.78 (0.22–2.69)	1.88 (0.93–3.81)	0.0137*
TRF1	0.54 (0.31–0.67)	0.93 (0.35–0.99)	1.00 (0.38–1.05)	0.251
TRF2	1.98 (1.21–2.89)	1.40 (0.82–1.79)	2.10 (1.02–3.11)	0.205
TIN2	3.24 (2.64–3.93)	2.13 (0.81–2.68)	2.23 (0.51–3.92)	0.172
TPP1	1.90 (0.96–2.24)	0.73 (0.40–3.23)	0.70 (0.70–0.78)	0.220
RAP1	3.68 (1.93–4.14)	1.89 (0.61–2.37)	3.86 (0.87–4.18)	0.077

Asterisk means there are significantly differences between untreated group, recovering group, and controls.

**Table 4:** Telomere length of SAA patients and controls.

Number	Ages (y)	Group	TRF (kb)
1	69	1	7.61
2	60	1	4.76
3	16	1	4.33
4	26	1	3.52
5	44	1	3.54
6	33	1	3.92
7	49	1	4.74
8	5	1	3.29
9	20	1	6.30
10	5	2	8.07
11	18	2	4.66
12	19	2	7.40
13	20	2	7.21
14	20	2	7.00
15	23	2	6.05
16	25	2	5.98
17	27	2	5.98
18	29	2	8.78
19	31	2	4.31
20	36	2	8.83
21	45	2	8.38
22	47	2	8.56
23	48	2	5.96
24	18	3	13.45
25	67	3	7.40
26	7	3	16.57
27	35	3	10.41
28	37	3	19.82
29	25	3	9.20
30	47	3	6.12
31	48	3	7.08
32	52	3	6.12
33	15	3	20.34

Group 1: untreated SAA patients (*n* = 9).

Group 2: recovering SAA patients (*n* = 14).

Group 3: controls (*n* = 10).
